# Metabolic characterization of the chitinolytic bacterium *Serratia marcescens* using a genome-scale metabolic model

**DOI:** 10.1186/s12859-019-2826-1

**Published:** 2019-05-06

**Authors:** Qiang Yan, Seth Robert, J. Paul Brooks, Stephen S. Fong

**Affiliations:** 1 Department of Chemical and Life Science Engineering, School of Engineering, Virginia Commonwealth University, West Hall, Room 422, 601 West Main Street, P.O. Box 843028, Richmond, VA 23284-3028 USA; 2 Department of Statistical Sciences and Operations Research, Virginia Commonwealth University, P.O. Box 843083, Richmond, VA 23284 USA; 3 Center for the study of Biological Complexity, Virginia Commonwealth University, Richmond, VA 23284 USA

**Keywords:** Genome-scale metabolic model, *Serratia marcescens*, 2,3-butanediol, *N*-acetylneuraminic acid, RNASeq, *n*-butanol, Chitin, Flux balance analysis

## Abstract

**Background:**

*Serratia marcescens* is a chitinolytic bacterium that can potentially be used for consolidated bioprocessing to convert chitin to value-added chemicals. Currently, *S. marcescens* is poorly characterized and studies on intracellular metabolic and regulatory mechanisms would expedite development of bioprocessing applications.

**Results:**

In this study, our goal was to characterize the metabolic profile of *S. marcescens* to provide insight for metabolic engineering applications and fundamental biological studies. Hereby, we constructed a constraint-based genome-scale metabolic model (*i*SR929) including 929 genes, 1185 reactions and 1164 metabolites based on genomic annotation of *S. marcescens* Db11. The model was tested by comparing model predictions with experimental data and analyzed to identify essential aspects of the metabolic network (e.g. 138 essential genes predicted). The model *i*SR929 was refined by integrating RNAseq data of *S. marcescens* growth on three different carbon sources (glucose, *N*-acetylglucosamine, and glycerol). Significant differences in TCA cycle utilization were found for growth on the different carbon substrates, For example, for growth on *N*-acetylglucosamine, *S. marcescens* exhibits high pentose phosphate pathway activity and nucleotide synthesis but low activity of the TCA cycle.

**Conclusions:**

Our results show that *S. marcescens* model *i*SR929 can provide reasonable predictions and can be constrained to fit with experimental values. Thus, our model may be used to guide strain designs for metabolic engineering to produce chemicals such as 2,3-butanediol, *N*-acetylneuraminic acid, and *n*-butanol using *S. marcescens*.

**Electronic supplementary material:**

The online version of this article (10.1186/s12859-019-2826-1) contains supplementary material, which is available to authorized users.

## Background

With recent advances in high-throughput data and bioprospecting, the breadth of novel and interesting biochemistry continues to expand. In terms of the large amount of data and resources available, one current challenge is to interpret raw data into knowledge that can help better understand complex biological systems. Genome-scale metabolic modeling (GSMM) provides gene-protein-reaction level specificity and can combine metabolic network with fundamental genomic and biochemical information. However, there are still a number of limitations [1]. For instance, because metabolic pathway fluxes are underdetermined, which means always existence of alternative flux states according to different pathway usage that generate an indistinguishable physiological phenotype. This is an underlying issue with GSMMs that limits the level of detailed predictions these models can make when running simulations (presence of alternate optimal solutions) if not additional data/information is used. In this study, we investigate the poorly-characterized chitinolytic bacterium, *Serratia marcescens*, and utilize RNAseq data to achieve a better understanding of its metabolic network.

*S. marcescens* is unique among enteric bacteria in many aspects. It secretes extracellular DNase, gelatinase, lipase, several proteases, a red pigment (prodigiosin), chitinases and a chitin binding protein. It is believed to be one of the most efficient chitin-degrading bacteria in the environment [2, 3]. *S. marcescens* Db11 contains ten chitinase-related proteins [4] leading to studies focused on various facets of the *S. marcescens* chitinolytic mechanisms [5–8]. Due to the high efficiency of processing chitin, several individual *S. marcescens* chitinase genes have been cloned into model bacterial species (e.g. *Escherichia coli*) [9–11, 46, 47]. The cloned enzymes were isolated in good concentrations but failed to show similar level of chitinolytic activity as is found in *S. marcescens*. This may be due to the complexity of chitin degradation systems and synergy between multiple enzymes [4, 8, 12, 13]. Hence, it is suggested that the best way to fully utilize the chitinolytic capabilities of *S. marcescens* may be developing *S. marcescens* rather than moving its chitinases into other systems by heterologous expression.

The ability to produce chemicals of industrial importance using inexpensive chitinolytic biomass has been a recent focus [14, 49, 50]. Microbial conversion of chitin waste into value-added chemicals, a similar concept as consolidated bioprocessing (CBP), can be socially and economically beneficial [14–16, 48]. For *S. marcescens* Db11, the sequencing of its genome in 2014 sets up a milestone towards understanding this industrially applicable microbe [17]. The initial developments in the characterization of *S. marcescens* are listed in Table 1. This is a promising development toward making use of the chitinolytic capabilities of this microbe to reduce the complex multi-step bioprocess to CBP.

**Table 1:** Significant milestone for *S. marcescens* research and characterization

Year	Development	Citation
1980	Genus Constructed	[45]
1991–1999	Physical Characterization	[48, 49]
1998	Shrimp/crab chitin degradation study to produce *N*-acetylglucosamine	[5]
2014	Genome sequenced	[17]
2017	Development of genetic modification method	[4]

In studying *S. marcescens* Db11, we believe that it can be developed into a facile chitinolytic system for consolidated bioprocessing. Recently, we established molecular tools for genetic engineering this organism using: 1) an in-frame gene deletion approach based on endogenous exonucleases by introducing linear DNA fragments and 2) a shuttle vector that contains functional replication origin and expression elements (e.g. promoter and RBS) [4]. These approaches allowed us to study the function of a chitinase regulator protein (ChiR) by characterizing physiological differences in a ChiR overeexpression strain and a ChiR deletion strain. ChiR is characterized as a positive regulator protein and is essential for chitinase production in *S. marcescens*. In the same study, the ChiR overexpression *S. marcescens* produced 1.13 g/L 2,3-butanediol from 2% crystal chitin. The ability to convert chitin into biochemicals makes this organism an interesting candidate for a chitin-based CBP [15, 18, 49]. Thus, our group aims to present a system level understanding of the metabolic network of *S. marcescens* to facilitate future engineering applications.

A reconstructed GSMM can provide a framework to understand cellular processes and can be used to find target for focused metabolic engineering to yield products of biotechnological value [19]. The most widely-used algorithms for design and simulation of genome-scale constraint-based metabolic models such as OptKnock [20], OptForce [21], EMiLio [22] are based on flux distribution and flow through chemically balanced reactions (FBA). Flux balance analysis (FBA) uses linear algorithm to optimize an objective function:

$$ {\displaystyle \begin{array}{c}\operatorname{Maximize}:\mathrm{Z}\\ {}\mathrm{Subject}\ \mathrm{to}:\mathrm{Sv}=0,\\ {}{a}_i\le {v}_i\le {b}_i\kern0.5em \mathrm{for}\ \mathrm{all}\ \mathrm{reactions}\kern0.5em i,\end{array}} $$

where, Z is the flux towards objective function, e.g. biomass production and production optimization, S is stoichiometry of the reactions as matrix, *v* is the reaction flux vector, *a*_*i*_ and *b*_*i*_ are the constraints based on the flux *v*_*i*_ of the reaction *i* [23].

After compiling biochemical information into a draft model, a second step of model curation can be done by integrating high-throughput omics data to realize “content in context” [23]. A variety of applications using incorporating omics data with the framework of GSMM have been reported for many prokaryotic microbes to more closely match cellular processes [24–26]. In this study, we aim at understanding metabolite profile of *S. marcescens* Db11 and its chitinolytic regulation systems. Therefore, a genome-scale metabolic model, *i*SR929, consisting of 1185 reactions, 1164 metabolites and 929 genes was constructed. The constructed model was analyzed for 1) accuracy compared to experimental results; 2) prediction of essential genes; 3) metabolic differences under different carbon growth conditions. We also proposed three potential chemical compounds for metabolic engineering implementation.

## Results

### Construction of *S. marcescens* GSMM

Based upon the genome sequence of *S. marcescens* Db11 [17] and available physiological evidence, a genome-scale constraint-based metabolic model for *S. marcescens*, hereafter denoted *i*SR929, was constructed. The reaction database used for drafting the model includes but is not limited to sources such as KEGG [27], BiGG [28], rBioNet [29], UniProt [30] and MBRole [31]. *i*SR929 contains 1185 reactions representing the function of 929 genes and 1164 metabolites (see Table 2). Metabolic gaps were identified and addressed guided by a computational gap-filling algorithm [19, 24, 25, 32, 33]. Among the 1100 gene associated reactions of the total 1185 reactions, there are 795 reactions that are associated with only one reaction and there are 305 reactions associated with more than one enzyme, meaning either isozymes or enzyme complexes. Of the 1164 metabolites, 1099 metabolites are intracellular, 43 metabolites are extracellular, and 22 metabolites are boundary. We have added gene-protein-reactions (ChiA, ChiB, ChiC, SMDB11_1994, Chb, NagZ, SMDB11_1542, and SMDB11_4602) that lead to converting chitin into *N*-acetylglucosamine. In addition, we have added ChiB as a deacetylase that can convert *N*-acetylglucosamine to glucosamine, and added NagZ as a glucosamine kinase that phosphorylates glucosamine to glucosamine-6-P. Thus, the model can indicate the biotransformation of chitin to glucosamine, and glucosamine can be a carbon source that enters into carbon central metabolism. Furthermore, we added chitobiose degradation reactions to the model based upon the annotation and experimental evidence (EC 3.2.1.52, SMDB11_0477, SMDB11_1190 SMDB11_1542 and SMDB11_4602).

**Table 2:** Overview of genome-scale constraint-based model of *S. marcescens i*SR929

	*Serratia marcescens*
Genome size	5.11 Mb
ORFs	4832
Included genes	929
Reactions associated with only 1 gene	795
Reactions associated with more than 1 gene	305
Reactions with gene associated	1185
Intracellular metabolites	1099
Extracellular metabolites	43
Boundary metabolites	22

**Fig. 1:**
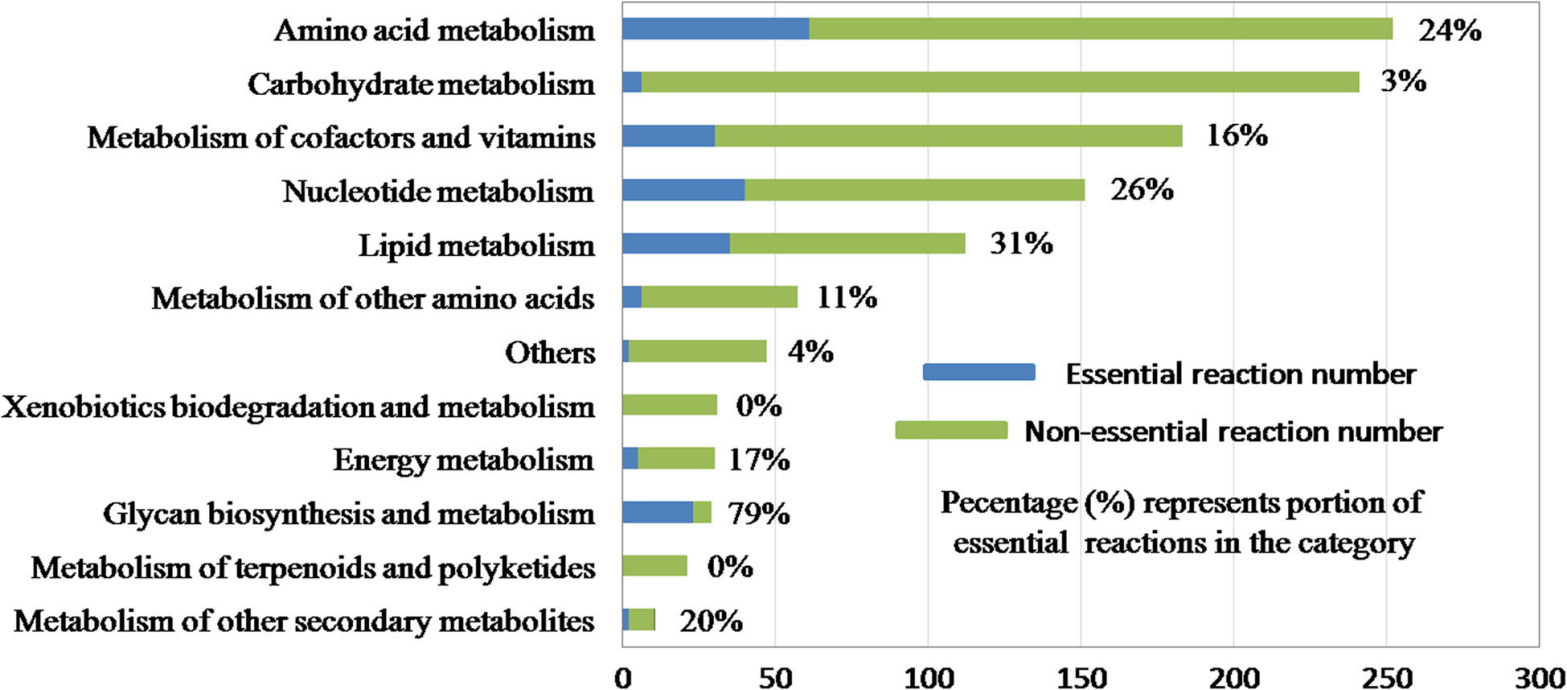
Reactions by functional category with number of reactions in model iSR929. The number of model-predicted essential genes in each category is shown in blue bar. The percentile of the model-predicted essential genes to its corresponding metabolism category is annotated on the right of each row

The breakdown of *i*SR929 by functional categories is represented in Fig. 1. Amino acid metabolism was the largest subsystem with 253 reactions. Experimentally, *S. marcescens* appears to have the capacity to synthesize all 20 amino acids [4], and *i*SR929 reflects this. Other large groups of reactions include subsystems related to carbohydrate metabolism (glycolysis, pentose phosphate pathway, pyruvate metabolism, and TCA cycle), cofactor and vitamin metabolism (nicotinate and nicotinamide metabolism, folate biosynthesis, and porphyrin and chlorophyll metabolism) and nucleotide metabolism (including reactions related to purine and pyrimidine synthesis). The model also includes reactions relating to the synthesis of lipids, including fatty acid, glycerophospholipid, and glycerolipid metabolism.

**Fig. 2:**
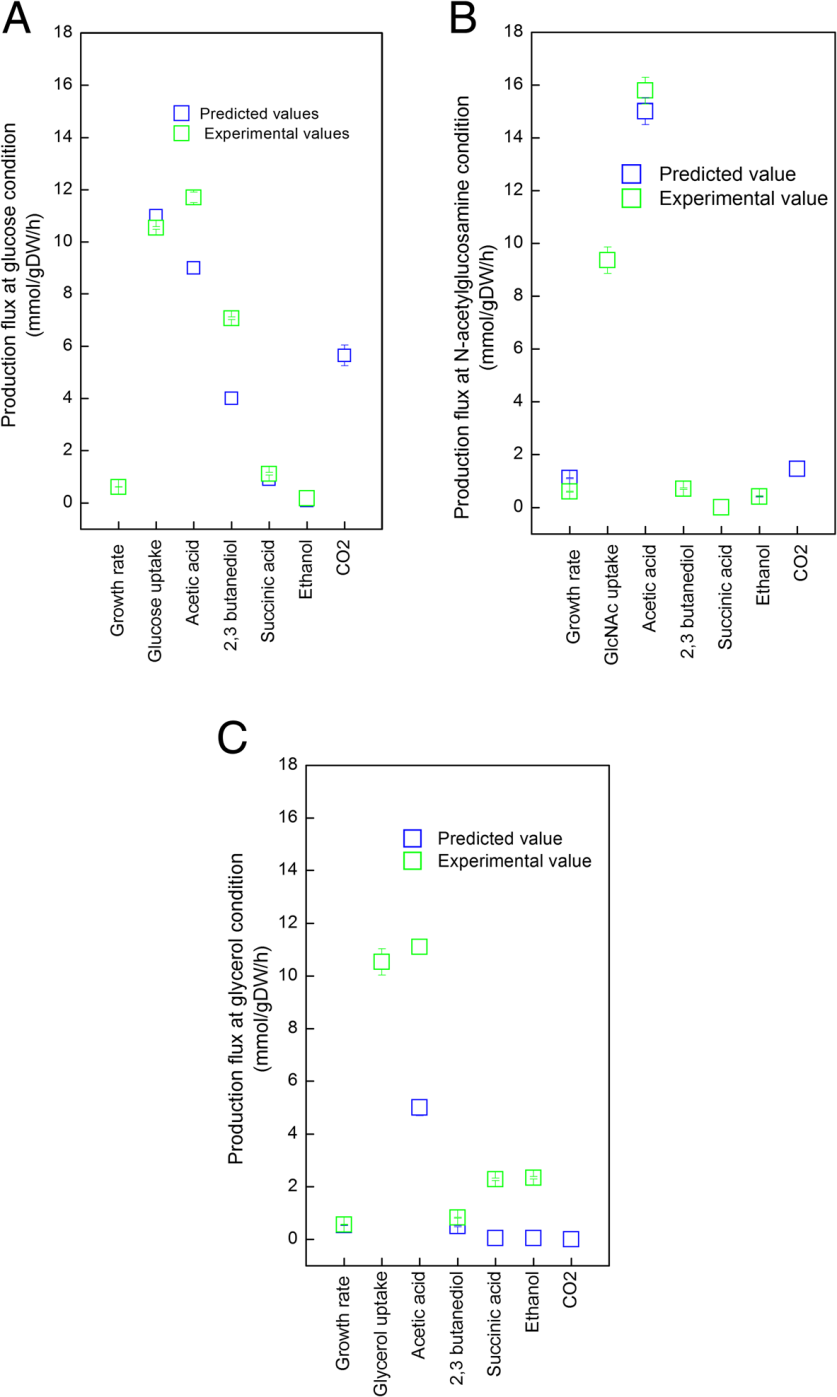
Comparison of model predictions to experimental values. *S. marcescens* iSR929 was used to simulate growth in multiple conditions. Actual and predicted flux rates are shown, and predicted fermentation product production rates are shown as ranges as determined by flux variability analysis. For each simulation, the boundary fluxes for growth rate, sugar uptake, acetic acid, 2,3-butanediol, succinic acid, ethanol were constrained to match the measured fluxes during **a** glucose, **b** *N*-acetylglucosamine, **c** glycerol conditions

To test the predictions of the *i*SR929 model, we simulated growth of *S. marcescens* by applying FBA, assuming minimal media conditions with one of three possible carbon sources (glucose, *N*-acetylglucosamine, or glycerol). We then compared model predictions to experimentally observed growth rates and fermentation product secretion profiles of *S. marcescens* grown in batch culture. In addition to the carbon source, the in silico minimal medium used for simulations contained water (H2O), ammonia (NH4), sulfate (SO4), phosphate (pi), calcium (Ca2), ferrous iron (Fe3), hydrogen sulfide (H2S), potassium (K), magnesium (Mg2), pantothenate (pnto-r), and nicotinate D-ribonucleotide (nmn). In each of the three simulation conditions (glucose, *N*-acetylglucosamine, and glycerol), we applied progressively more experimentally determined constraints associated with by-product secretion rates to determine how closely the computational results could match the experimental results given the possibility of alternate optimal solutions. Figure 2 shows the simulation results for each growth condition when exchange rates for the carbon sources, acetic acid, and succinic acid were constrained to match experimental observations.

### Computational prediction of gene knockout targets

A comprehensive analysis of in silico single gene deletions were conducted with *i*SR929 using glucose as a carbon source and the other minimal media components (as described above), and constraining the glucose uptake rate to its experimentally observed value for batch growth (10.53 mmol/gDW/h). Gene essentiality results are shown in Fig. 1. In the case of growth on glucose, we found that 138 (14.9%) of *S. marcescens* genes included in *i*SR929 were predicted to be essential. We also examined which subsystems of *i*SR929 contained the highest percentage of essential reactions (‘vulnerable subsystems’). Among the most vulnerable subsystems are amino acid metabolism, nucleotide metabolism, lipid metabolism, and metabolism of cofactors and vitamins.

### mRNA sequencing of *S. marcescens* under growth on different carbon sources

mRNA samples of *S. marcescens* Db11 were prepared from cell cultures under four conditions: M9 medium with glucose, M9 medium with *N*-acetylglucosamine, M9 medium with glycerol, and LB medium. Overall, the average coverage per gene (4831 genes) (see Additional file 1: Table S1) implies a high-depth-coverage (generally 250).

### Integrating RNASeq data into the GSMM

The integration of RNAseq data of *S. marcescens* grown in M9 medium with glucose, *N*-acetylglucosamine, or glycerol was conducted using the *i*SR929 model and a mixed integer linear programming algorithm approach [34]. In order to establish a cutoff value of gamma (lower bound cutoff), we initially categorized gene expression level into 58 cutoffs based on an exponential of 1.2, and a distribution of gene numbers against each gene expression level range was then plotted, a representative figure of M9 glucose condition was shown in Additional file 1: Figure S1. The lower bound is 3.00, which excludes around 10% of the total number of genomic genes across all the conditions, and the upper bound is 850.56, shown in Additional file 1: Table S2.

Simulations were then run on the *S. marcescens* model after transcriptomic data integration for the three growth conditions (M9 glucose, M9 *N-*acetlyglucosamine, M9 glycerol) separately, where a reaction flux is computationally constrained to 1000 if the corresponding gene’s RPKM is over 850.56 and a reaction flux is 0 if the gene’s RPKM is less than 3.00. Raw data of reaction flux can be found in Additional file 1: Table S3 and a summary of the active metabolic reactions for the three growth conditions were categorized in different metabolic pathway modules (Table 3). When growing on glucose, there were 150 active reactions. Among these 150 reactions, the most highly represented functional categories were: carbohydrate metabolism (42), amino acid metabolism (38), lipid metabolism (12), cofactor metabolism (12), and nucleic acid metabolism (6). When growing on *N*-acetylglucosamine, there were 132 active reactions. Among these 132 active reactions, the majority of the reactions were: carbohydrate metabolism (39), amino acid metabolism (28), lipid metabolism (6), cofactor metabolism (9), and nucleic acid metabolism (10). When growing on glycerol, there were 146 active reactions. Among these 146 active reactions, the functional categories were: carbohydrate metabolism (44), amino acid metabolism (41), lipid metabolism (6), cofactor metabolism (8), and nucleic acid metabolism (7).

**Table 3:** Numbers of active metabolic reactions in *i*SR929 by running simulation based on the transcriptomic data

	Glucose	*N*-acetylglucosamine	Glycerol
Carbohydrate metabolism			
Butanoate metabolism	4	4	2
Citrate cycle (TCA cycle)	13	0	10
Glycolysis / Gluconeogenesis	7	10	9
Glyoxylate and dicarboxylate metabolism	3	2	1
Pentose and glucuronate interconversions	0	2	2
Pentose phosphate pathway	6	14	11
Propanoate metabolism	1	0	1
Pyruvate metabolism	6	5	6
Amino sugar and nucleotide sugar metabolism	2	2	2
Amino acid metabolism			
Alanine, aspartate and glutamate metabolism	4	3	5
Arginine and proline metabolism	9	10	23
beta-Alanine metabolism	0	0	2
Glutathione metabolism	2	2	0
Taurine and hypotaurine metabolism	2	2	2
Valine, leucine and isoleucine biosynthesis	5	2	2
Glycine, serine and threonine metabolism	16	9	7
Nucleic acid metabolism			
Purine metabolism	6	1	6
Pyrimidine metabolism	0	9	1
Lipid metabolism			
Fatty acid degradation	2	0	0
Glycerophospholipid metabolism	8	4	4
Alpha-Linolenic acid metabolism	2	2	2
Energy metabolism			
Oxidative phosphorylation	3	3	4
Nitrogen metabolism	0	0	1
Metabolism of cofactors and vitamins			
Pantothenate and CoA biosynthesis	3	1	0
Riboflavin metabolism	2	2	2
One carbon pool by folate	6	6	6
Biosynthesis of other secondary metabolites			
Monobactam biosynthesis	2	2	2
Streptomycin biosynthesis	2	2	2
Xenobiotics metabolism			
Benzoate degradation	1	0	0
Nitrotoluene degradation	2	2	2
Transportation	31	31	29
Total	150	132	146

**Fig. 3:**
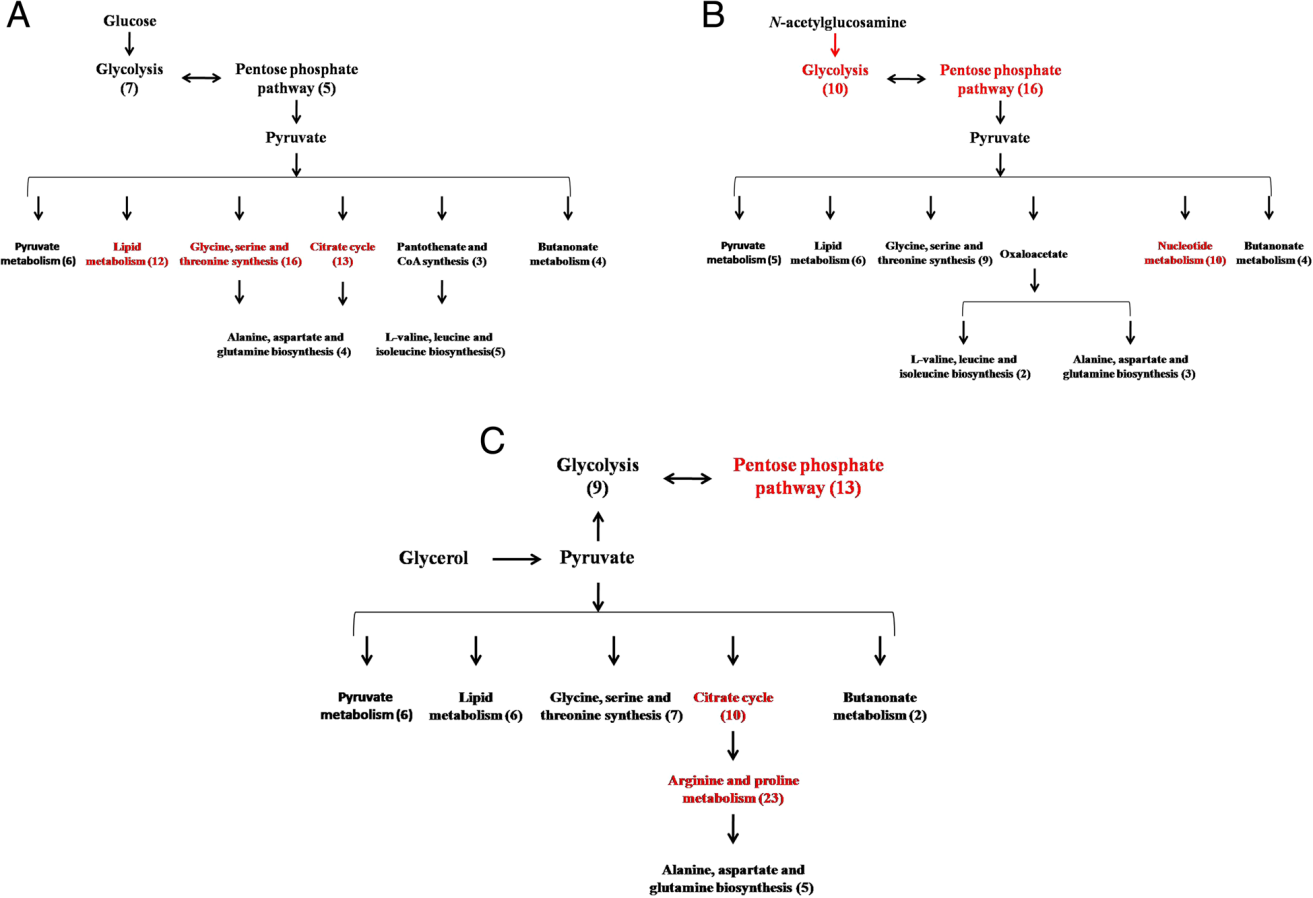
Summary of active pathways after running Ruppin algorithm using model iSR929 under each growth condition. **a** Growth on M9 glucose; **b** growth on M9 *N*-acetylglucosamine; **c** growth on M9 glycerol. The model-predicted active metabolism pathways were marked in red

The main active pathways for the metabolism of glucose as a sole carbon source were shown in Fig. 3a. After transport, glucose input is routed through glycolysis and pentose phosphate pathway to yield pyruvate. Then, the main flux goes to the TCA cycle, butanoate metabolism (yielding 2,3-butanediol), amino acid synthesis, lipoprotein synthesis, and pyruvate metabolism (yielding ethanol, formate, lactate, and acetate).

The main active pathway for the metabolism of *N*-acetylglucosamine as a sole carbon source was shown in Fig. 3b. For the metabolism of *N*-acetylglucosamine, *S. marcescens* is believed to phosphorylate transported *N-*acetylglucosamine by a membrane type II phosphotransferase (SMDB11_0473). The *SMDB11_0473* gene expression level in the *N*-acetylglucosamine condition (RPKM 967.20) was 12.09- and 5.13-fold than those under glucose (RPKM 80.23) and glycerol (RPKM 188.36) conditions, respectively, supporting that *N-*acetylgluosamine is likely phosphorylated intracellular. The carbon flows through glycolysis metabolism from fructose-6P to regenerate glucose-6P. The glucose-6P flows to the pentose phosphate pathway to yield pyruvate. Then, the majority of the carbon flux was distributed into purine and pyrimidine metabolism, butanoate metabolism (secreted 2,3-butanediol as an end-product), pyruvate metabolism (yielding ethanol, formate, lactate, and acetate as end-products), amino acid synthesis, and lipoprotein synthesis.

The main active pathways for the metabolism of glycerol as a sole carbon source were shown in Fig. 3c. Glycerol metabolism is thought to be converted to pyruvate by three steps: a membrane facilitator protein (SMDB11_4021), an ATP-dependent glycerol kinase (SMDB11_4022), and a glycerol-3-phosphate dehydrogenase (SMDB11_3886) [35]. The SMDB11_4021 expression level in the glycerol growth condition (RPKM 1772.81) was 89.5- and 70.8-fold higher than those of glucose and *N*-acetylglucosamine condition. The *SMDB11_4022* expression level in the glycerol condition (RPKM 4535.25) was 76.6- and 63.6-fold higher than those of glucose (RPKM 59.20) and *N*-acetylglucosamine (RPKM 71.28) condition. The gene expression level of *SMDB11_3886* in the glycerol condition (RPKM 1683.89) was 59.9- and 54.7-fold higher than those of glucose (RPKM 28.10) and *N*-acetylglucosamine (RPKM 30.78) condition. The main active flux goes to glycolysis, pentose phosphate pathway, amino acid synthesis, and pyruvate metabolism.

### *S. marcescens* exhibits low activity in TCA cycle with *N*-acetylglucosamine as a sole carbon source

**Fig. 4:**
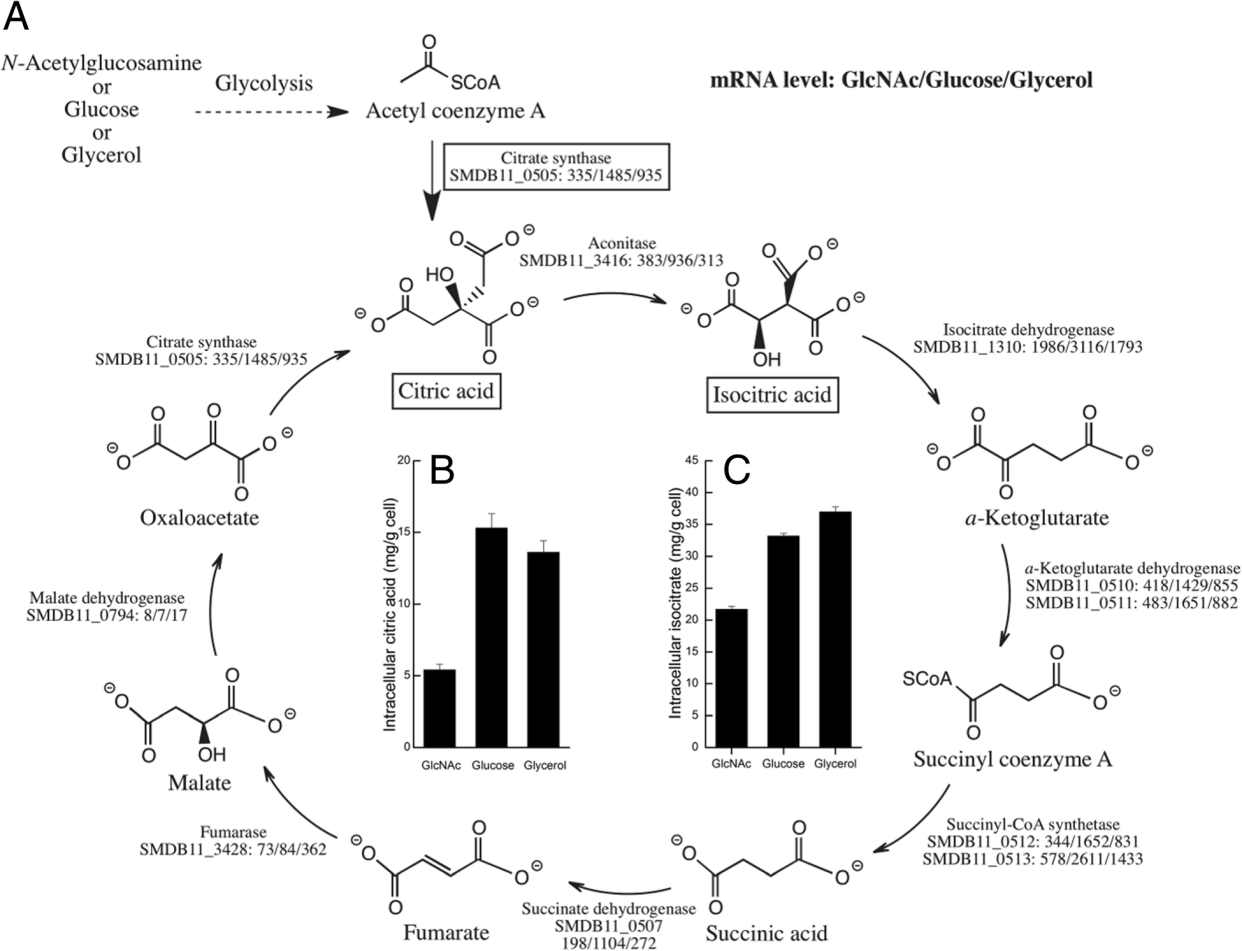
**a** Mapping of TCA cycle metabolism pathway genes mRNA level under *N*-acetylglucosamine, glucose or glycerol condition. **b** Intracellular citric acid concentration of *S. marcescens* Db11 when growing on *N*-acetylglucosamine, glucose or glycerol. **c** Intracellular isocitric acid concentration of *S. marcescens* Db11 when growing on *N*-acetylglucosamine, glucose or glycerol

According to model simulation results, we have examined metabolic fluxes differences in the carbon central metabolism, namely, glycolysis, pentose phosphate pathway, and TCA cycles, shown in Additional file 1: Figure S2A, S2B, and Fig. 4, respectively. The main metabolic differences among the three carbon sources are found in the TCA cycle. When glucose is the sole carbon source, the TCA cycle is fully active; under glycerol condition, the TCA cycle is partially active (R00361_smac, R01082_smac, R02164_smac, R00405_smac, R02570_smac, and R00621_smac); while under *N*-acetylglucosamine condition, the TCA cycle is not active. The gene expression level of the TCA cycle pathway under *N*-acetylglucosamine, glucose or glycerol condition were mapped in Fig. 4a. In general, the mRNA level of the genes in the TCA cycle support the model prediction results: compared to mRNA level of the TCA cycle genes under *N*-acetylglucosamine condition, those corresponding genes under glucose or glycerol condition are generally higher. In particular, the *SMDB11_0505* gene, which encodes a citric acid synthase that converts acetyl-CoA to citric acid, may play the key role in determining the model prediction since under glucose or glycerol condition, the *SMDB11_0505* mRNA level are both above the high threshold, while under *N*-acetylglucosamine condition, the *SMDB11_0505* mRNA level is below the high threshold.

To test for differences in TCA cycle flux, measurement of intracellular citric acid and isocitric acid levels can be used as indicators of TCA cycle activity [36]. Experimental measurement of intracellular citric acid and isocitric acid concentrations for growth on *N*-acetylglucosamine were lower than those for growth on glucose or glycerol, providing some support for the predicted lower TCA cycle flux results (Fig. 4b and c). Additionally, when *N*-acetylglucosamine is the sole carbon source, *S. marcescens* appears to utilize intermediates (e.g. oxaloacetate, fumarate, and 2-oxoglutarate) as precursors for amino acids synthesis, and synthesis of nucleotides (e.g. purine and pyrimidine) comes from the ribose-5P generated from the pentose phosphate pathways since *N*-acetylglucosamine is an ideal carbon source for amino-sugar and nucleotides synthesis (e.g. C:N ratio 8:1).

### Cofactor metabolism and energy ATP metabolism

Based on metabolic flux analysis predicted by the model, the common consumption/re-generation of NADH under the three carbon source conditions are supported in the amino acid metabolism, butanoate metabolism and pentose phosphate metabolism; the common consumption/re-generation of NADPH under the three carbon source conditions are supported from the glycolysis and gluconeogenesis metabolism, one carbon pool by folate, and transport; common pathways associated with ATP consumption and generation under the three-carbon source conditions are supported from amino acid metabolism, one carbon pool by folate, pyruvate metabolism and transportation of molecules, shown in Additional file 1: Table S4.

When growing *S. marcescens* under *N*-acetylglucosamine as a sole carbon source, NAD+/NADH tends to be a main cofactor compared to NADP/NADPH. It is worth noting that under *N*-acetylglucosamine as a sole carbon source condition, additional NADH cylces can be achieved in the pyrimidine metabolism, and pentose and glucuronate interventions by a nucleotide phosphorylase and a 3-dehydro-L-gulonate 2-dehydrogenase. This is in agreement with the observance shown in the carbon metabolism map that nucleotide metabolism and pentose phosphate pathways are highly active when using *N*-acetylglucosamine. Particular ATP consumption and generation pathways in *S. marcescens* under *N*-acetylglucosamine condition include glycine, serine and threonine metabolism and pyrimidine metabolism.

## Discussion

### Metabolic engineering applications of *S. marcescens* using the model *i*SR929

*S. marcescens* possesses interesting native metabolic capabilities (active chitinolytic system and native production of 2,3-butanediol) and can potentially be engineered for biochemical production. Based on the aforementioned experimental observations and computational simulation results, we selected three chemical compounds (2,3-butanediol, *n*-butanol, and *N*-acetylneuraminic acid) as potential production targets and illustrate pathway designs for the production of each.

**Fig. 5:**
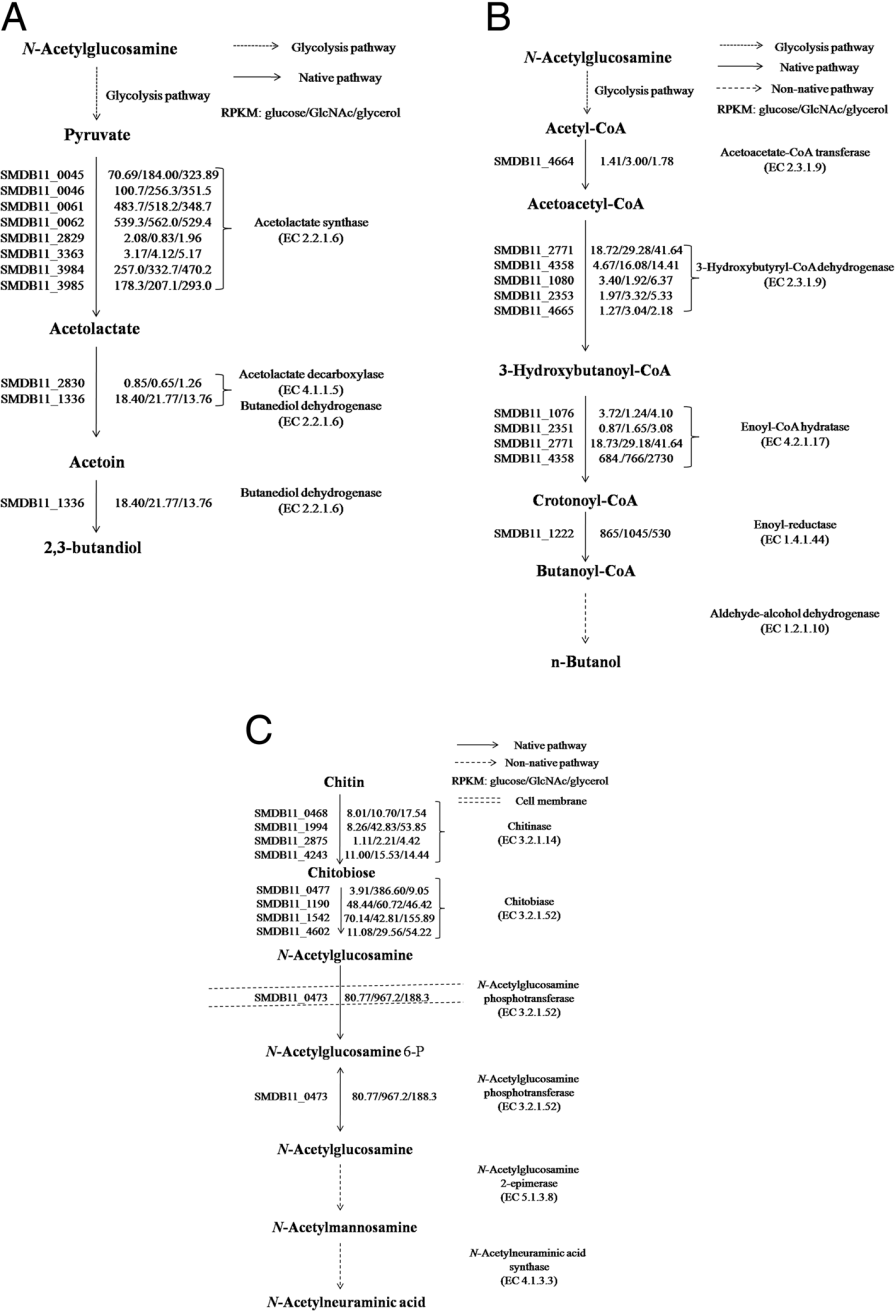
Proposed pathways for potential chemicals of interest production as metabolic engineering targets by *S. marcescens* and their corresponding gene expression values obtained from RNAseq data. **a** 2,3-butenadiol **b** *n*-butanol **c** *N*-acetylneuraminic acid

*S. marcescens* Db11 is a native 2,3-butanediol producer. Direct production of 2,3-butanediol from crystal chitin by *S. marcescens* Db11 have been experimentally validated and the model *i*SR929 shows 2,3-butanediol production across all growth conditions [4]. The proposed pathways for production of 2,3-butanediol and the corresponding annotated genes are shown in Fig. 5a. Unlike other reported *S. marcescens* 2,3-butanediol producers [37, 38], *S. marcescens* Db11 harbors only two 2,3-butanediol dehydrogenases (meso and (2S,3S)) and relatively small amount of (2S,3S)-2,3-butanediol dehydrogenase was expressed compared to the meso-2,3-butanediol dehydrogenase, indicating meso-2,3-butanediol is the major product.

The second target of interest is a biofuel chemical, *n*-butanol. The *i*SR929 incorporates most of the reactions present in the butanoate metabolism as inferred from genomic and biochemical data. Besides the 2,3-butanediol synthetic pathway, two active reactions (R01171 and R01975) were found under glucose growth conditions and one active reaction (R01171) was observed under glycerol growth conditions. One consideration of the computational simulation is that butanoate is a product of a secondary pathway and *S. marcescens* may not overproduce genes associated with this pathway during normal exponential growth. Thus, it is not necessarily surprising that growth simulations of a wild-type strain with no genetic manipulation may not show flux through the butanoate metabolism pathways. The proposed *n*-butanol synthetic pathway and associated gene expression levels in *S. marcescens* Db11 are shown in Fig. 5b. The annotated pathway for *n*-butanol production is partially complete but is missing the enzyme required for the last reaction (EC 1.2.1.10 alcohol dehydrogenase). A bifunctional alcohol dehydrogenase (AdhE2) from *Clostridium acetobutylicum* is reported with highest activity of alcohol production [39] and can be an appropriate enzyme candidate for heterologous expression in *S. marcescens*. Addition of an alcohol dehydrogenase would theoretically enable production of *n*-butanol, but it is also likely that additional pathway optimization would be necessary to improve flux through the necessary reactions as expression of the remaining endogenous butanoate genes is relatively low (e.g. SMDB11_4664 acetoacetate-CoA transferase).

The third chemical target of interest is *N*-acetylneuraminic acid which is not natively produced by *S. marcescens* Db11. Compared to glucose-based *N*-acetylneuraminic acid synthesis (demonstrated in *E. coli* [40]), use of *N*-acetylglucsaomine/chitin as a carbon source has the following advantages: 1) it requires only two steps for *N*-acetylneuraminic acid synthesis from *N*-acetylglucosamine compared to glucose and 2) no additional nitrogen source is needed [15, 41]. Growth of *S. marcescens* on *N*-acetylglucosamine showed that the enzyme (SMDB11_0473) associated with uptake of *N*-acetylglucosamine is highly active (RPKM 967.20). After transport, SMDB11_0473 (*N*-acetylglucosamine transferase) is a reversible *N*-acetylglucosamine transferase that can generate intracellular *N*-acetylglucosamine [40], shown in Fig. 5c. Intracellular *N*-acetylglucosamine can be converted to *N*-acetylneuraminic acid by additional two biochemical reactions (*N*-acetylmannosamine 2-epimerase and *N*-acetylneuraminic acid synthase) [40]. Further overproduction of *N*-acetylneuraminic acid may require carefully balance the heterologous pathways since heterologous expression may generate metabolic burden [42].

## Conclusion

In this study, we constructed a genome-scale metabolic model, *i*SR929, for the chitinolytic bacterium *Serratia marcescens*. The model contains 929 genes, 1185 reactions and 1164 metabolites. Initial experimental testing showed that the iSR929 model is able to reasonably predict quantitative phenotypes for *S. marcescens*. Integrating transcriptomic data for growth on different carbon sources (e.g. glucose, *N*-acetylglucosamine and glycerol) to the model provides insight into metabolic difference that are condition-dependent including different utilization of the TCA cycle for growth on *N*-acetylglucosamine. Three biochemical targets (2,3-butanediol, *n*-butanol, and *N*-acetylneuraminic acid) were proposed as potential metabolic engineering implementation targets and pathway activities were given. Overall, our results provide insight into the metabolic function of *S. marcescens* and reveal potential targets for engineering *S. marcescens* for biochemical production.

## Methods

### Microbial growth

*Serratia marcescens* Db11 was purchased from the Caenorhabditis Genetics Center (Twin City, USA http://www.cbs.umn.edu/CGC) [43]. The *S. marcescens* strain was grown in M9 medium or LB medium supplemented with various carbon sources. Carbon sources in the M9 medium were used at the following concentrations: glucose, 5 g/L; *N*-acetylglucosamine, 5 g/L; glycerol 5%. *S. marcescens* pre-cultures were grown in LB medium at 30 °C and 250 rpm overnight. 2.5% seed culture was inoculated at 50 mL M9 minimum medium with a 250 mL Erlenmeyer flask at 30 °C, initial pH 7.5, and 220 rpm for growth experiments. Stock cultures of *S. marcescens* were maintained at − 80 °C in 26% (v/v) glycerol.

### Construction of the model *i*SR929

The core *S. marcescens* GSMM is a compile of metabolic reactions occurring in *S. marcescens*, compiled based on biochemical information from genome annotations [17] and experimental evidence. A draft model of initial biochemical reactions was constructed based on gene reading frames that encode enzymes with predicted functions in the genomic annotations available from IMG, UniProt, and KEGG [27, 30, 44]. Specifically, Enzyme Commission (EC) numbers of annotated *S. marcescens* genes were adapted to select reactions from a set of database reactions.

### Flux balance analysis (FBA)

The FBA simulations were run using in-house python scripts based on the linear programming algorithm according to previous publications [19, 24, 25, 32, 33].

To this end, no *Serratia* biomass equation is available; therefore, we modified *E. coli* biomass equation by including components that are essential for *Serratia* growth [51–53]. Detailed biomass equation can be found as follows:

$$ 0.05\ 5\mathrm{mthf}+5.0\mathrm{E}-5\ \mathrm{accoa}+0.488\ \mathrm{ala}\_\mathrm{L}+0.0010\ \mathrm{amp}+0.281\ \arg \_\mathrm{L}+0.229\ \mathrm{asn}\_\mathrm{L}+0.229\ \mathrm{asp}\_\mathrm{L}+45.7318\ \mathrm{atp}+1.29\mathrm{E}-4\ \mathrm{clpn}\_\mathrm{SM}+6.0\mathrm{E}-6\ \mathrm{coa}+0.126\ \mathrm{ctp}+0.087\ \mathrm{cys}\_\mathrm{L}+0.0247\ \mathrm{datp}+0.0254\ \mathrm{dctp}+0.0254\ \mathrm{dgtp}+0.0247\ \mathrm{dttp}+1.0\mathrm{E}-5\ \mathrm{fad}+0.25\ \mathrm{g}\mathrm{ln}\_\mathrm{L}+0.25\ \mathrm{g}\mathrm{lu}\_\mathrm{L}+0.582\ \mathrm{g}\mathrm{ly}+0.154\ \mathrm{g}\mathrm{ly}\mathrm{cogen}+0.203\ \mathrm{g}\mathrm{tp}+45.5608\ \mathrm{h}2\mathrm{o}+0.09\ \mathrm{h}\mathrm{i}\mathrm{s}\_\mathrm{L}+0.276\ \mathrm{i}\mathrm{le}\_\mathrm{L}+0.428\ \mathrm{le}\mathrm{u}\_\mathrm{L}+0.0084\ \mathrm{lps}\_\mathrm{SM}+0.326\ \mathrm{ly}\mathrm{s}\_\mathrm{L}+0.146\ \mathrm{met}\_\mathrm{L}+0.00215\ \mathrm{nad}+5.0\mathrm{E}-5\ \mathrm{nad}\mathrm{h}+1.3\mathrm{E}-4\ \mathrm{nad}\mathrm{p}+4.0\mathrm{E}-4\ \mathrm{nad}\mathrm{p}\mathrm{h}+0.001935\ \mathrm{p}\mathrm{e}\_\mathrm{SM}+0.0276\ \mathrm{p}\mathrm{e}\mathrm{p}\mathrm{tido}\_\mathrm{SM}+4.64\mathrm{E}-4\ \mathrm{p}\mathrm{g}\_\mathrm{SM}+0.176\ \mathrm{p}\mathrm{h}\mathrm{e}\_\mathrm{L}+0.21\ \mathrm{p}\mathrm{r}\mathrm{o}\_\mathrm{L}+5.2\mathrm{E}-5\ \mathrm{p}\mathrm{s}\_\mathrm{SM}+0.035\ \mathrm{p}\mathrm{trc}+0.205\ \mathrm{s}\mathrm{e}\mathrm{r}\_\mathrm{L}+0.0070\ \mathrm{s}\mathrm{p}\mathrm{md}+3.0\mathrm{E}-6\ \mathrm{s}\mathrm{u}\mathrm{ccoa}+0.241\ \mathrm{thr}\_\mathrm{L}+0.054\ \mathrm{trp}\_\mathrm{L}+0.131\ \mathrm{tyr}\_\mathrm{L}+0.0030\ \mathrm{u}\mathrm{dpg}+0.136\ \mathrm{u}\mathrm{tp}+0.402\ \mathrm{val}\_\mathrm{L}-->45.5608\ \mathrm{adp}+45.56035\ \mathrm{h}+45.5628\ \mathrm{p}\mathrm{i}+0.7302\ \mathrm{p}\mathrm{p}\mathrm{i}+\mathrm{Biomass} $$

### Gap analysis

FBA-GAP was used to fill metabolic gaps where reactions are missing in the initial reconstructed model. The gap-filling was conducted according to methods described previously [19, 24, 25].

### Essentiality

Constraint-based models can be used to analyze in silico gene essentiality through running FBA [45]. Briefly, using the GPR relationships, the effect of an in silico gene knockout on reaction activities is assessed by assuming a gene is deleted or non-functional. If a gene is in silico deleted, that reaction is constrained to “zero” flux to simulate the effect of the gene deletion. Then, the model with the new constraint is analyzed by FBA, maximizing flux using the growth objective for batch growth on glucose. If the maximum flux of the biomass reaction is “zero”, the deleted gene is determined as an essential gene. On the contrary, if the maximum flux of the biomass reaction is over “zero”, the deleted gene is determined as a non-essential gene. The process is repeated for the next cycle to analyze another gene by resetting all the constraints back to default values. The essentiality analysis was conducted using M9 minimal medium with glucose as carbon source.

### mRNA sequencing

The total RNA of *S. marcescens* Db11 was extracted from cells harvested during mid-log phase. A QIAGEN (Venlo, Netherlands) RNeasy Mini kit along with the proper RNA protect reagent was used. Then, the mRNA was isolated, enriched, and reverse transcribed into cDNA. The sequencing of cDNA was conducted using paired-end reads using Illumina Hiseq 2500 (San Diego, CA). The detailed information of the RNASeq data can be found in a previous publication [49]. In addition, the completed gene transcriptional levels of *S. marcescens* are listed in Additional file 1: Table S1.

### Measurement of cell density

The measurement of *S. marcescens* culture density was generally quantified at OD600 using a Biomate3 UV/VIS spectrophotometer (Thermo Fisher Scientific, Waltham, MA).

### Quantification of secreted metabolites

The concentration of *N*-acetylglucosamine, glucose, glycerol and all metabolic end-products (acetic acid, succinic acid, 2,3-butanediol, and ethanol) were analyzed by HPLC described in details in our previous publication [49].

### Integration of RNASeq data with genome-scale reconstruction

The integration of RNASeq data with the *i*SR929 model was analyzed using a mixed integer linear programming algorithm approach [34], In brief, gene expression states were determined according to a gene expression value (g) vs. an arbitrary threshold value (γ), shown as follows:

$$ \mathrm{Gene}\kern0.17em \mathrm{expression}\kern0.17em \mathrm{state}=\left\{\begin{array}{cc}\hbox{-} 1& \mathrm{g}=0\\ {}0& 0<\mathrm{g}\le \upgamma \\ {}1& \upgamma <\mathrm{g}\end{array}\right. $$

Where g is the relative expression level as determined by RNAseq (e.g. RPKM) and γ is the threshold value that was manually set. The resulting gene states were then integrated into *i*SR929 using the gene-protein-reaction (GPR) relationships to generate lists of reactions predicted to be high or low fluxes [34].

### Determination of intracellular citric acid and isocitric acid concentration

Intracellular levels of citric acid and isocitric acid were evaluated using a citrate assay kit (Sigma-Aldrich, St. Louis, MO) and an isocitric acid kit (BioVision, Milpitas, CA), respectively. In brief, *S. marcescens* cell growth was stopped at 2.5 h (OD600 about 0.4) and cells were collected by centrifuging at 4 °C, 15,000 rpm, and 10 min. Cell pellets were lysed using a commercial detergent BugBuster (Millipore Sigma, Temecula, CA). Citric acid and isocitric acid assays were conducted according to the manual’s instruction. 10 μL supernatant samples were added in a total volume of 100 μL reaction. Citric acid and isocitric acid concentrations were determined at 570 nm and 450 nm by a VERSAmax microplate reader (Molecular Devices, Sunnyvale, CA), respectively.

## Additional files

Additional file 1: **Table S1.** Average RPKM values of *S. marcescens* gene expression level at three different growth conditions. **Table S2.** Number of genes excluded/included after the lower/upper bound cutoff. **Table S3.** *S.marcescens i*SR929 reaction flux values after running Ruppin algorithm. **Table S4.** NAD+/NADH associated reactions under three carbon source conditions. **Figure S1.** Representative figure of gene expression level distribution under M9 glucose medium growth condition. **Figure S2.** Relative gene expression levels of reactions in the metabolic map of (A) Glycolysis (B) Pentose Phosphate Pathway of *S. marcescens*. (ZIP 898 kb)

Additional file 2: Model file. (TXT 116 kb)

Additional file 3: Biomass objective function file. (TXT 1 kb)

Additional file 4: Exchange file. (TXT 2 kb)

Additional file 5: GPR file. (TXT 199 kb)

## Abbreviations

5mthf: 5-methyltetrahydrofolate
accoa: Acetyl-CoA
ala_L: L-alanine
arg_L: L-arginine
asn_L: L-asparagine
asp_L: L-asparatate
clpn_SM: Cardiolipin (Serratia)
cys_L: L-cystidine
gln_L: L-glutamine
glu_L: L-glutamic acid
gly: Glycine
GSMM: Genome-scale metabolic model
his_L: L-histidine
ile_L: L-isoleucine
leu_L: L-leucine
lps_SM: Lipopolysaccharide *Serratia*
lys_L: L-lysine
met_L: L-methionine
pe_SM: phosphatidylethanolamine *Serratia*
peptido_SM: Peptidoglycan subunit of *Serratia*
pg_SM: Phosphatidylglycerol *Serratia*
phe_L: L-phenylalanine
pi: Phosphate
ppi: Diphosphate
pro: L-proline
ps_SM: Phosphatidylserine *Serratia*
ptrc: Putrescine
ser_L: L-serine
spmd: Spermidine
succoa: Succinyl-CoA
thr_L: L-threonine
trp_L: L-tryptophan
tyr_L: L-tyrosine
udpg: UDP-glucose
val_L: L-valine
