# Supplementary material for: Metabolic characterization of the chitinolytic bacterium Serratia marcescens using a genome-scale metabolic model
Source: BMC Bioinformatics. 2019 May 6;20:227. doi: 10.1186/s12859-019-2826-1 (PMC6501404; doi:10.1186/s12859-019-2826-1)
Supplement: Supplementary file 1 — Table S1. Average RPKM values of S. marcescens gene expression level at three different growth conditions. Table S2. Number of genes excluded/included after the lower/upper bound cutoff. Table S3. S.marcescens iSR929 reaction flux values after running Ruppin algorithm. Table S4. NAD+/NADH associated reactions under three carbon source conditions. Figure S1. Representative figure of gene expression level distribution under M9 glucose medium growth condition. Figure S2. Relative gene expression levels of reactions in the metabolic map of (A) Glycolysis (B) Pentose Phosphate Pathway of S. marcescens. (ZIP 898 kb) [file 12859_2019_2826_MOESM1_ESM.zip › supplementary information.docx]

**Supplementary information:**

**Journal:** BMC Systems Biology

**Title:** **Metabolic characterization of the chitinolytic bacterium *Serratia marcescens* using a genome-scale metabolic model**

Qiang Yan^1^, Seth Robert^1^, J Paul Brooks^2,3^, and Stephen S Fong^1,3,*^

Authors and affiliations

^1^Department of Chemical and Life Science Engineering, Virginia Commonwealth University, Richmond, VA 23284, USA

^2^Department of Statistical Sciences and Operations Research, Virginia Commonwealth University, Richmond, VA 23284, USA

^3^Center for the study of Biological Complexity, Virginia Commonwealth University, Richmond, VA 23284, USA

^*^Dr. Stephen Fong, Virginia Commonwealth University, School of Engineering, Department of Chemical and Life Science Engineering West Hall, Room 422, 601 West Main Street, P.O. Box 843028, Richmond, Virginia 23284-3028; [ssfong@vcu.edu](mailto:ssfong@vcu.edu); (804)827-7038

**Table S1** Average RPKM values of *S. marcescens* gene expression level at three different growth conditions (see xlsx file).

**Table S2** Number of genes excluded/included after the lower/upper bound cutoff.

| Number of genes | M9 glucose | M9 *N*-acetylglucosamine | M9 glycerol |
| --- | --- | --- | --- |
| Lower bound 3.00 | 500 | 459 | 196 |
| Upper bound 850.56 | 251 | 271 | 314 |

**Table S3** *S.marcescens* *i*SR929 reaction flux values after running Ruppin algorithm (see xlsx file).

**Figure S1** Representative figure of gene expression level distribution under M9 glucose medium growth condition


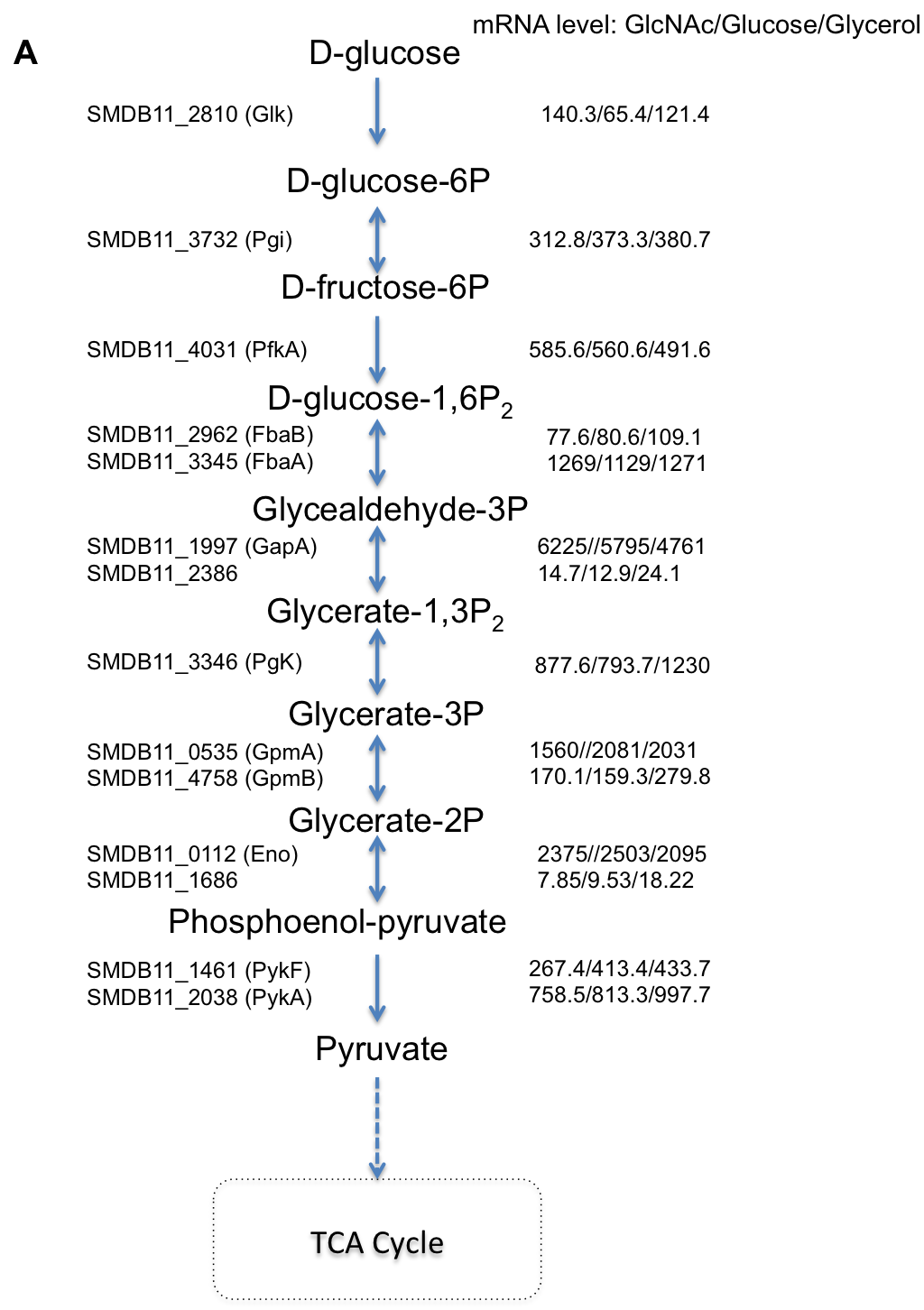


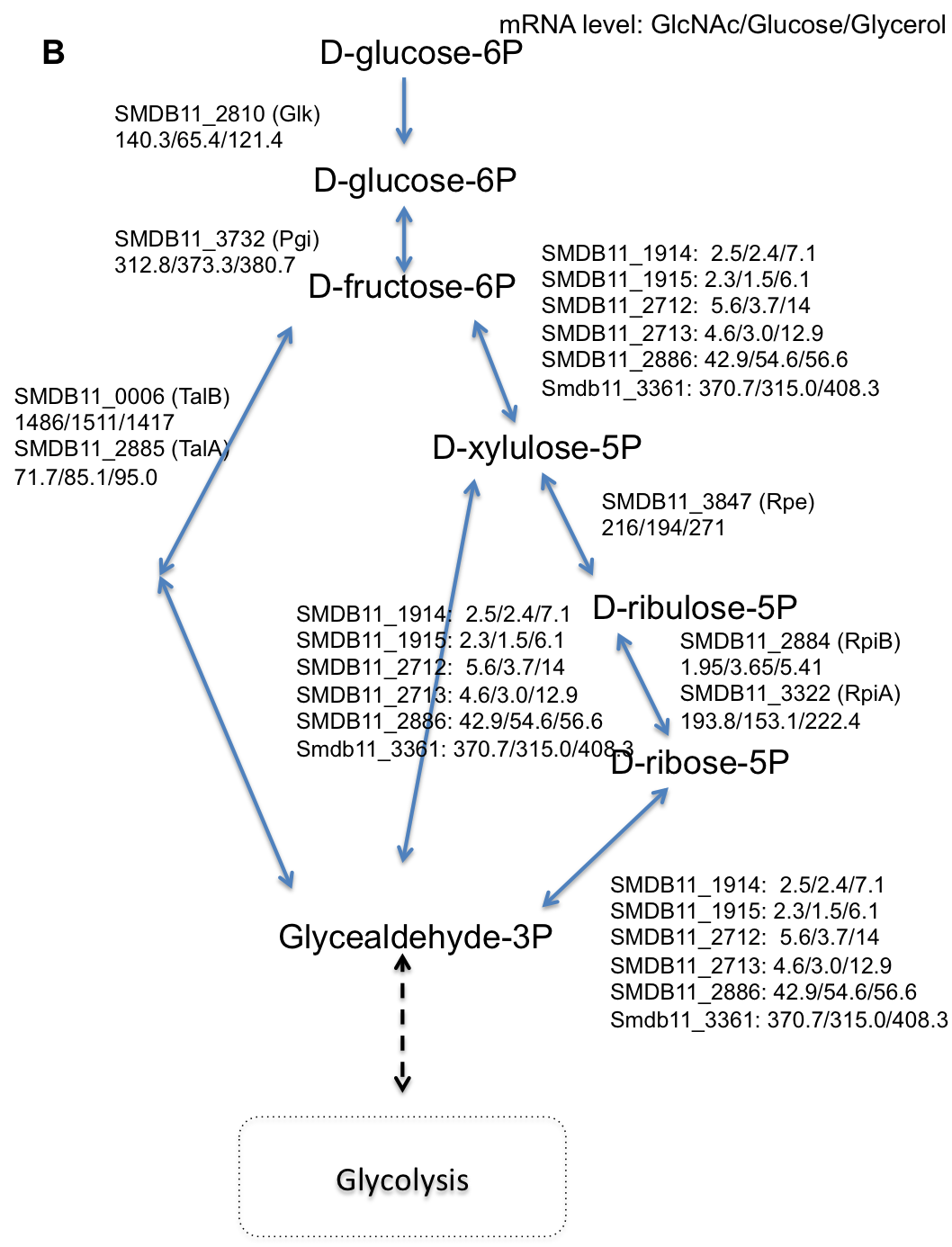


**Figure S2** Relative gene expression levels of reactions in the metabolic map of (A) Glycolysis (B) Pentose Phosphate Pathway of *S. marcescens*.
